# Supplementary material for: Iron Dyshomeostasis in COVID-19: Biomarkers Reveal a Functional Link to 5-Lipoxygenase Activation
Source: Int J Mol Sci. 2022 Dec 20;24(1):15. doi: 10.3390/ijms24010015 (PMC9819889; doi:10.3390/ijms24010015)
Supplement: Supplementary file 1 [file ijms-24-00015-s001.zip › ijms-2050394-supplementary.pdf]

Supplementary Materials of:

Iron dyshomeostasis in COVID-19: biomarkers reveal a functional link to 5-lipoxygenase activation

Beatrice Dufrusine [1, 2, 3], Silvia Valentinuzzi [2,4], Sandra Bibbò [2, 3], Verena Damiani [2, 3], Paola Lanuti [3, 5], Damiana Pieragostino [2, 3], Piero Del Boccio [3, 4], Ersilia D'alessandro [3], Alberto Rabottini [3], Alessandro Berghella [1], Nerino Allocati [2], Katia Falasca [5, 6], Claudio Ucciferri [6], Francesco Mucedola [6], Marco Di Perna [7], Laura Martino [7], Jacopo Vecchiet [5, 6], Vincenzo De Laurenzi [2, 3 *], and Enrico Dainese [1 *]

1 Department of Bioscience and Technology for Food Agriculture and Environment, University of Teramo, 64100 Teramo, Italy
2 Department of Innovative Technologies in Medicine and Dentistry, University "G. d'Annunzio" of Chieti-Pescara, 66100 Chieti, Italy
3 Center for Advanced Studies and Technology (CAST), University "G. d'Annunzio" of Chieti-Pescara, 66100 Chieti, Italy
4 Department of Pharmacy, University "G. d'Annunzio" of Chieti-Pescara, 66100 Chieti, Italy
5 Department of Medicine and Aging Science, "G. d'Annunzio" University of Chieti-Pescara, 66100 Chieti, Italy
6 Clinic of Infectious Diseases, S.S. Annunziata Hospital, 66100 Chieti, Italy
7 Pneumology Department, "SS Annunziata" Hospital, 66100 Chieti, Italy
* Correspondence: delaurenzi@unich.it (V.D.L.); edainese@unite.it (E.D.); Tel.:+39-0861-266876 (E.D.)

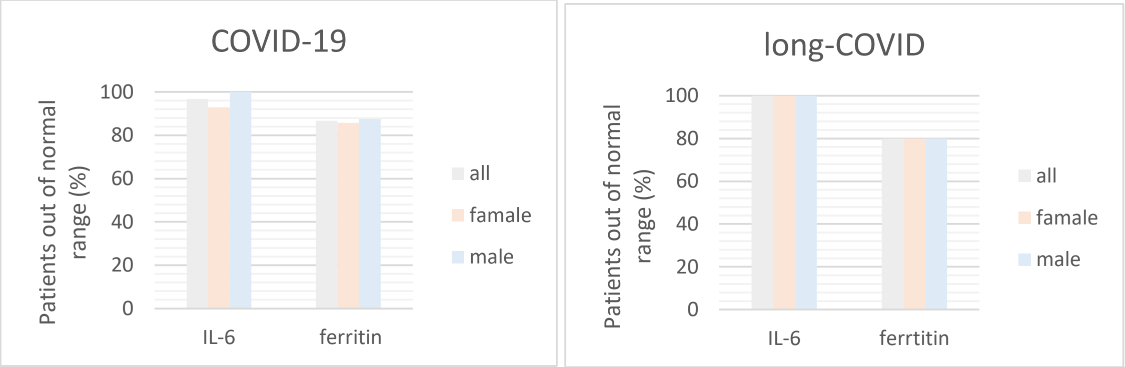

Figure S1. Percentage of patients out of normal range for IL-6 and ferritin data, for all patients and according to gender in COVID-19 and long-COVID group.

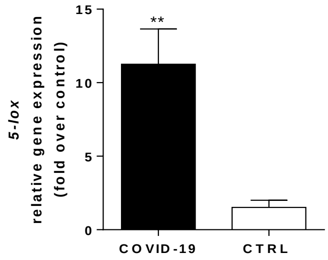

Figure S2. Gene expression levels of *5-lox* in a nasopharyngeal swab of COVID-19 patients (n=15) were analyzed using quantitative RT-PCR. Data reported in this figure are the mean ± SE of two independent experiments (**$p<0.01$).

The bioinformatical tool Ingenuity Pathway Analysis (IPA) highlighted via "Diseases and Functions" analysis the downstream involvement of an oxidative stress-related biological function, named "Metabolism of hydrogen peroxide", which turns out to be associated with high statistical significance as stated by low overlap p-values in both comparisons of lymphocytes protein cargo between COVID-19 patients and controls ($1.5 \cdot 10^{-8}$ and $1.7 \cdot 10^{-10}$, respectively for CD19+ and CD3+ lymphocytes). The prediction of the activation state by z-score estimation suggests an overall trend of upregulation of the function mentioned above for both CD19+ and CD3+ lymphocytes in Covid-19 patients versus controls, however the prediction is shown to be associated with statistical significance only in CD19+ cells (z-score in CD19+ lymphocytes from the comparison patients/controls: 2.6; z-score in CD3+ lymphocytes from the comparison patients/controls: 0.7). The Supplementary Figure 3 shows the upregulation of "Metabolism of hydrogen peroxide" in CD19+ lymphocytes from COVID-19 patients versus healthy controls, as qualitatively displayed by the orange color of the function at the bottom of the interaction network, which appears to be justified and predicted by the differential expression of the specific dataset proteins above.

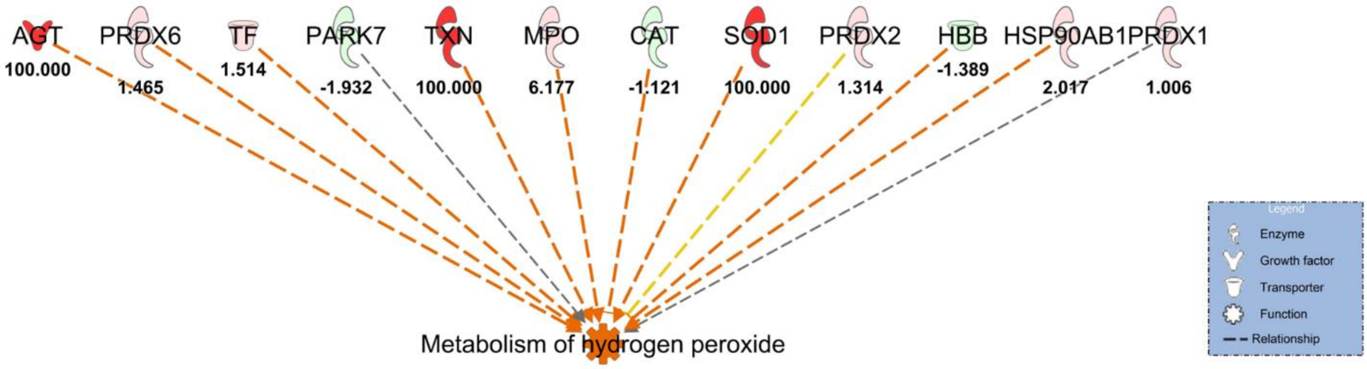

Figure S3. Downstream activation of metabolism of hydrogen peroxide in CD19+ lymphocytes from Covid-19 patients versus controls. Numbers below each dataset protein represent the protein expression ratios. For dataset proteins, reddish colors stand for increased measurements, greenish colors for decreased ones. Orange arrows indicate a predicted activated relationship, yellow arrows display inconsistent findings with the state of the downstream function, grey arrows represent a not predicted effect.

Detection	Fluorochrome	Vendor	Ab Clone	Catalog	Amount for test
CD3	APC	BD Biosciences	HIT3a	555342	10 µl
CD19	BV421	BD Biosciences	HIB19	562441	5 µl

Table S1. List of reagents Keys: Allophycocyanin (APC); Brilliant Violet (BV).
